# Woman focused smoking cessation programming: a qualitative study

**DOI:** 10.1186/s12905-016-0298-2

**Published:** 2016-03-12

**Authors:** Nadia Minian, Jessica Penner, Sabrina Voci, Peter Selby

**Affiliations:** Addictions Program, Centre for Addiction and Mental Health, 100 Stokes Street, Toronto, ON M6J 1H4 Canada; Department of Family and Community Medicine, University of Toronto, 500 University Avenue, Toronto, ON M5G 1V7 Canada; Department of Psychiatry, University of Toronto, 250 College Street, 8th floor, Toronto, ON M5T 1R8 Canada; Dalla Lana School of Public Health, University of Toronto, 155 College Street, Toronto, ON M5T 3M7 Canada; Ontario Tobacco Research Unit, 155 College Street, Toronto, ON M5T 3M7 Canada

**Keywords:** Smoking, Tobacco use disorder, Smoking cessation, Women

## Abstract

**Background:**

Several studies of smoking cessation programs in clinical settings have revealed poorer outcomes for women compared to men, including counselling alone or in combination with pharmacotherapy. The objective of the current study was to explore treatment and program structure needs and preferences among female clients in a specialized smoking cessation clinic in an academic mental health and addiction health science centre in order to inform program design so that it meets the needs of female clients.

**Methods:**

Four focus groups were conducted with current and former female clients (*n* = 23, mode age range = 50–59 years old, 56.5 % were still smoking and 43.5 % had quit) who had registered for outpatient smoking cessation treatment. Questions were designed to examine what aspects of the services were helpful and what changes they would like to see to better assist them and other women with quitting smoking. A thematic analysis of the raw data (audio recordings and notes taken during the focus groups) was conducted using a phenomenological theoretical framework.

**Results:**

Themes that emerged indicated that females trying to quit smoking are best supported if they have *choice* from a variety of services so that treatment can be individualized to meet their specific needs; psychosocial *support* is provided both one-one-one with health care professionals and by peers in support groups; *free pharmacotherapy* is available to eliminate financial barriers to use; *women-specific educational topics and support groups* are offered; the clinic is *accessible* with evening/weekend hours, options to attend a local clinic, and childcare availability; and *communication* about clinic services and operation are clear, readily available, and regularly updated.

**Conclusions:**

An ideal smoking cessation program for women includes a women’s centred approach with sufficient variety and choice, free pharmacotherapy, non-judgmental support, accessible services and clear communication of program options and changes. Findings may suggest an actionable list of adaptations that can be adopted by other clinics providing smoking cessation services to women.

**Electronic supplementary material:**

The online version of this article (doi:10.1186/s12905-016-0298-2) contains supplementary material, which is available to authorized users.

## Background

Several smoking cessation studies of programs in clinical settings have revealed poorer outcomes for women compared to men across various types of interventions, including counselling of varying intensity alone or in combination with pharmacotherapy [1, 2]. However, data from population-level surveys suggest this finding does not generalize to all women [3]. For example, large-scale national surveys in the UK, USA and Canada suggest that while older women exhibit lower quit rates compared to men, the opposite is true for women below 50 [3]. Conflicting findings across studies highlight the diversity amongst women as a group, some of whom are likely to experience greater difficulty with quitting, dependent on factors such as intervention type and treatment setting.

Regardless of whether overall success rates differ between men and women, there is evidence that both sex (biology) and gender (social) play important roles in smoking cessation [2]. With relation to sex, researchers have found that hormone changes related to the menstrual cycle can have an impact on smoking cessation [4, 5] and that nicotine replacement therapy is less effective for women than for men [6], possibly because women derive greater reinforcement from non-nicotinic aspects of smoking [7, 8]. With regards to gender, researchers have shown that women tend to report lower levels of motivation and confidence related to quitting [9], and greater fears related to post-cessation weight gain [10, 11]. In addition, conditions that are probably caused (in part) by the interaction of sex and gender, such as psychiatric co-morbidities (including depression and anxiety), have been also shown to play a role in smoking cessation [4, 12, 13]. Women are twice as likely to suffer from depression than men [14], thus it is an important factor to consider in smoking cessation treatment for women. Women may also experience difficulties surrounding post-pregnancy relapse [15–17]. These sex and gender differences suggest that a “one-size-fits-all” approach may not be effective [18].

While several clinicians and researchers have tailored smoking cessation programs with the goal of improving outcomes for women, a recent systematic review of programs targeted to women failed to yield conclusive findings that could inform the design of smoking cessation programming to fully account for the needs of women [19] who had sought treatment in a smoking cessation clinic to inform program design. Evidence from a subpopulation of clients enrolled in the clinic [20] revealed that female clients exhibited significantly lower quit rates compared to male clients (unpublished data) and this was the impetus for the current study. We report on the findings from a series of focus groups with women who had sought treatment in the clinic to seek feedback regarding what features of treatment they had experienced as helpful and supportive of their quit attempts and areas for change or improvement in the future.

## Methods

### Setting

All female clients had sought treatment in an outpatient smoking cessation clinic in Toronto, Ontario that provides individualized treatment to both women and men. A variety of services are provided by an inter-professional team, including: support groups, educational workshops, brief individual counselling, pharmacotherapy (nicotine replacement therapy [NRT], bupropion, and varenicline), and self-help materials. Clients seeking treatment in this clinic on average tend to be more heavily nicotine dependent, older, less educated, have a lower income, and report a greater number of previous unsuccessful quit attempts than the general population of smokers in Ontario [21]. Close to half of those who seek treatment in the clinic are women.

### Participants

A total of 204 female clients aged 18 years or older who had received services in the clinic between June 2012 and June 2013 were eligible for the study. In an attempt to have a full age range represented in the sample, the list of potential participants was sorted into several age groups (18–29; 30–39; 40–49 50–59; 60–60; ≥70) and telephone calls were made to females randomly selected within each group to invite participation. Telephone calls were made by a research assistant who had not had any previous contact with clients. Of the 160 women we attempted to contact, 99 could not be reached and 21 declined to participate. The 40 females who initially agreed to participate completed a short questionnaire over the telephone to confirm eligibility and to obtain a brief smoking history. Of these, 23 female clients ultimately attended and participated in one of the four focus groups (FG1 to FG4). Female clients were recruited until no new themes were evident during discussion, as occurred during the fourth focus group, and saturation was thus considered to have been achieved. The number of participants in each focus group ranged from 3 to 8. At the time of recruitment, 13 (56.5 %) reported they were still smoking and 10 (43.5 %) had quit. In addition, 14 (60.8 %) were current clients and 9 (39.1 %) were former clients. see Table 1 for additional demographic characteristics. Participants received a $25 CAD honorarium for participation and were reimbursed for the value of public transit travel expenses ($6 CAD). Due to the fact that the project was conducted for the purpose of quality improvement of clinical services, it was exempted from requiring ethics approval from the local institutional research board at the Centre for Addiction and Mental Health. All participants provided written consent before the start of the focus group; the consent form included a description of the study aims, methods, anticipated benefits and potential risks, confidentiality, and the right to refuse consent or withdraw from the study at any time without any negative consequences. In order to protect confidentiality, all identifying information was removed from the collected materials and names were not used when reporting findings. Participants were also encouraged to keep the meeting confidential.

**Table 1:** Participant demographics

	Number	Percent
Age group		
18–29	2	8.7
30–39	1	4.3
40–49	2	8.7
50–59	9	39.1
60–69	6	26.1
70+	3	13.0
Length of time in treatment at the clinic^a^		
< 1 month	2	8.7
1–3 months	1	4.3
3–6 months	10	43.5
6–12 months	3	13.0
> 1 year	7	30.4
Change in smoking since enrolled in treatment		
No change	2	8.7
Quit smoking	10	43.5
Quit and relapsed	3	13.0
Reduced smoking	6	26.1
Increased smoking	2	8.7

^a^Time in treatment is approximate and calculated based on the date (month) of attendance at an initial orientation session as well as the most recent visit to the clinic, as reported by the client at the time of recruitment

### Focus groups

We selected focus group methodology because it permitted a more exploratory examination of our research question than was possible with survey data, while also providing the flexibility to examine topics that arose in greater depth. For this reason, our questions were often more broad (see Additional file 1) so as to encourage the participants themselves versus the researchers to determine what topics were discussed. Focus groups also allowed us to determine what ideas or suggestions raised were supported by the larger group and thus were greater priorities for future implementation. The focus groups took place between July and August 2013. Both focus group moderators were female non-clinical staff that did not work in the clinic; each had previous experience conducting focus groups (NM: 10 years; JP: 1.5 years). The purpose of the focus groups was described to participants as seeking feedback from female clients to help improve services at the smoking cessation clinic. Participants were asked about past quit attempts, challenges they had faced when trying to quit smoking, their thoughts and experiences regarding the services at the clinic, and suggestions for what can be changed in order to improve services. Most questions were designed to elicit personal experiences and opinions from the female clients without being explicitly gender-specific in their phrasing. Focus groups were audio-recorded using a digital recorder (but not transcribed) and a research student took notes electronically during the discussion.

### Analysis

Focus group data was examined using thematic analysis within a phenomenological theoretical framework in order to investigate the lived experience of female clients who sought treatment for smoking cessation at the clinic [22, 23]. Both audio-recordings and notes were used for the analysis. The audio-recordings were used to both confirm and elaborate upon the content of the notes and also provided information regarding non-verbal communication (i.e., emphasis, tone, pauses, etc.) and the notes helped to fill in any gaps where recordings were inaudible. A single coder (JP) analyzed the recordings and notes to identify themes using an inductive process as follows: review of the raw data (recordings and notes); generating codes; identification of recurring themes; arrangement of the initial codes according to theme; and interpretation of each theme. Participant quotations were selected to illustrate each theme and are included throughout the text. Quotations were selected during review of the audio-recordings after themes were identified and were transcribed verbatim. A second analyst (SV) not blind to the original coding followed the same process to identify themes that emerged in the raw data. Any discrepancies were discussed until consensus was achieved. Both coders were not clinicians and had no contact with the participants aside from the conduct of the focus groups.

## Results

The following themes emerged during the focus groups, reflecting aspects of smoking cessation treatment that female clients perceived as helpful: choice, free pharmacotherapy, support, accessibility, communication, and women-specific topics and groups. Preferred clinic features are summarized in Table 2 according to theme.

**Table 2:** Important clinic features for women

Theme	Clinic features
Choice	A flexible, client-centred approach that can be tailored individually to meet specific needs
	Variety of services – options to participate in support groups, individual counselling, educational workshops/sessions, pharmacotherapy and self-help materials/tools
Free pharmacotherapy	Offer pharmacotherapy at no cost to eliminate financial barriers
Support	A non-judgmental, understanding atmosphere
	Support groups
	Peer support opportunities
	Opportunity to receive further support from a clinician outside of clinic setting/hours (e.g., telephone)
Accessibility	Evening and/or weekend hours
	Easily accessible clinic locations
	Childcare
Communication	Communication regarding programming options that is clear, readily available and regularly updated
	Multiple strategies to increase communication and reach more clients
Women-specific options	Provide information on women-specific topics relevant to smoking (e.g., smoking cessation and pregnancy or menopause, etc.)
	Female-only support groups as an option in addition to mixed-gender groups

### Choice

Participants strongly favoured an adaptive, client-centred approach which allowed treatment to be tailored to meet their specific needs. Their ability to choose from a variety of services (including psychosocial and pharmacotherapy) was viewed as necessary to meet the diverse needs of female clients and important for success.

*I like it more tailored to me because we started going one route but we found that, the doctor and I, that it wasn’t working… If I had just been given a prescription and told to go home, see you in 3 months…it wouldn’t have worked.* FG4

Though each client had their own preferences, all treatment options were considered helpful by the group as a whole. Participants also reported the benefits of receiving self-help materials and tools which they could use on their own time, such as a diary to chart their tobacco use.

*I think it’s a pretty synergistic package. So, the one on one with an MD, the groups, and the education workshops and modules. I think they all work together and I wouldn’t value one of them over another.* FG3

### Free pharmacotherapy

An important benefit of treatment at this clinic for many of the participants was the ability to receive free NRT. While it was cited as helpful in removing cost as a barrier to using NRT, the fact that there was a maximum of 6 months (within a 12-month period) provided was cited as stressful for some. Participants stated that some clients did not feel ready to taper off NRT at the end of their 6 months, and some may delay a quit attempt due to concern that they would not make good use of their limited supply if not successful. While the participants recognized the importance of the weaning process and “not becoming dependent on something else” (FG1), it was suggested that exceptions to the 6-month limit should be made on a case-by-case basis for some who need extended assistance.

*I’m afraid of running out of my patches because I can’t afford to do it and I’m afraid of wasting when I’m not ready.* FG1

### Support

Support from staff and fellow clients was cited as particularly helpful to the quitting process. Clients felt the clinic was a non-judgmental environment where they felt understood, in contrast to attitudes some participants had encountered outside the clinic from both loved ones and health care professionals alike. Participants expressed that in the clinic they never felt like they had failed if they were not able to stick to their quit plan, but instead practitioners and other clients continued to offer a very supportive, encouraging environment that helped motivate them to persist with their quit attempt.

*I really feel like for the first time these people [in the clinic] understand, they’re not judging you, and that’s so important because so often you are [judged] – “Why are you smoking? Just stop” – and that was a big help.* FG1

Support groups were described as an important component of treatment for many clients. Words such as “big motivator”, “fantastic” and “my lifeline” were used to describe these groups. Reasons for finding these groups helpful included: feeling accountable to others, strengthening motivation, reinforcing what had been learned, learning strategies that helped others successfully quit, and allowing those who quit to share their experience and be a role model for others.

*For somebody coming in, they might look at me being [quit for] 5 months, oh, maybe feel a little intimidated … but I would like to say to them, hang in there, because I was once like you. I got my inspiration from the lady next to me who had quit for 5 months at that time, so I wanted to be where she was, so that gave me the hope to go further.* FG3

While most viewed having individuals at different stages of the quit process together in the same group as beneficial, there was also some interest in groups being divided according to quit status (e.g., ex-smokers only). Not having pre-determined topics in the general support group (versus topic-specific groups) was felt to allow for the discussion to be flexible and responsive to the clients’ needs.

While the clinic was considered a supportive environment, some clients wanted more frequent support, such as through follow-up telephone calls or having the ability to call to talk to a clinician from the program when needed. In addition to receiving support, a few clients were interested in volunteering to offer support to others, such as through a buddy program.

### Accessibility

For some women the hours of operation and location of the clinic were cited as barriers to accessing services. Though many were able to visit the clinic during the day, many found daytime hours inconvenient because of the need to take time off work. Adding evening and weekend hours was recommended to make services more accessible. It was also suggested that start times for weekday evening group sessions take into account time needed to commute from work and eat dinner rather than start at 5 pm.

*I can’t get to the support groups ‘cause they don’t work with my schedule for work and I feel… like… [attending groups] would help.* FG1

Clients living far from the clinic’s downtown location indicated that the availability of services closer to home would make it easier to attend appointments.

*I think I didn’t come [to group] because it’s too far, the drive … Even when we leave here, all I’m thinking about is rush hour. I’m going to be stuck in rush hour… I’m far more inclined to go if it’s around the corner.* FG3

An additional suggestion made by some clients to improve accessibility of the clinic for women was to offer childcare services.

*For women especially… [it would be helpful] if it would be more accessible after hours and daycare would be HIGHLY appreciated.* FG1

### Communication

Clear communication was mentioned as being very important. Participants did not always know about or fully understand all of the services being offered, and believed that communication about services should be clear, readily available, and regularly updated.

*At the outset there should be a little outline. Here is the program…* FG3

*The front desk is the first point of contact… if they could keep hard copy calendars, so when somebody comes in… to see any of the staff, that there is a calendar for the up and coming months and what’s running… the other thing is maybe an option, I have email, I would like to have a calendar sent to me, so that I could you know kinda wiggle my schedule and maybe fit more in.* FG2

Some participants who were no longer in treatment said they would have benefitted from being contacted by the clinic when they either cancelled or failed to show for an appointment. A follow-up call might have been enough to encourage them to continue with treatment in the clinic.

### Women-specific topics and groups

Female clients suggested that it would be beneficial if some educational sessions covered women-specific topics, such as smoking cessation during menopause or during pregnancy. Opinions were mixed on whether a female-only group would be beneficial. Many did not think it made a difference if the groups had men or not, however, some thought it would be beneficial and felt more comfortable in a female-only support group.

*I would like to see, not gender-specific programs or anything like that, but, maybe some workshops specifically for women who have stopped smoking or are still smoking… maybe have some information available on things that impact women… specifically women’s health…* FG2

*If I’m talking about my period starting that morning and I’m cranky and I’m in pain, the guy next to me, he’s just not going to get it, he can try, he just won’t understand… sometimes it can be more comfortable…* FG4

## Discussion

The focus groups highlighted a number of aspects of smoking cessation treatment that female clients perceived as helpful: choice from multiple treatment options; free pharmacotherapy; non-judgmental support from staff and fellow clients; accessibility including evening and weekend hours and multiple clinic locations; clear communication regarding programming options; and women-specific topics and group sessions. The following discussion highlights how these results can be used to improve a clinic’s ability to meet women’s needs.

While it was evident that overall female clients valued pharmacotherapy and psychosocial support, there were differences between women in the exact type and combination of services they preferred. Having a range of options and the ability to choose was considered beneficial. Participants viewed individualized treatment as better able to meet the diverse needs of clients compared to standard treatment. Based on these findings, further research should test whether individualized programming approaches are associated with greater quit rate success for women compared to standardized programs.

A non-judgmental, supportive environment helped ensure that women did not feel a sense of failure if they had not met their quit goals and helped encourage them to continue with their quit plan. Those not comfortable with attending support groups were able to receive one-on-one support from a healthcare professional. In addition to support groups, there was indication that expanding services to include more formalized peer support opportunities would be beneficial for both women who have quit and those currently in process. Helping facilitate peer connections between interested clients could also be used as a means of increasing accessibility to support, as some women were interested in having support available outside of traditional appointments or hours of operation. Developing a multi-part workshop series for women could simultaneously provide information on issues related to smoking cessation that are specific to women while also creating an opportunity to create increased community among female clients. Women-centred programming often addresses the need for women-centred communication and interaction [24], thus we postulate that access to these supports may lead to improved quit rates for women. Women-centred information sheets are also in development in our clinic to address some of the specific topics raised by focus group participants, such as menopause and smoking.

Free NRT was frequently cited as helpful and an important component of treatment. While 26 weeks is much greater than most standard courses of NRT, it was evident that placing a limit on the amount provided added stress to the quitting process for some clients. While clients recognized the importance of tapering off the NRT, they felt that some exceptions should be made for individuals who required extended support. This might be a particular concern for women; meta-analysis has shown that short-term treatment gains made using NRT diminish more rapidly for women than men [6]. Further investigation is required to determine if some women would improve their quit rates with an extended course of NRT. Research has suggested that women may also require adjunct nonpharmacological support given evidence that smoking for women may be less reinforced by nicotine itself and more so by non-nicotine stimuli associated with smoking (e.g., sight and smell of cigarette smoke) that may not be addressed in treatment [7]. Mention of such external non-nicotine factors did not arise in the focus group discussions as a theme, highlighting the need for program providers to fully consider and balance what women are voluntarily able to identify as helpful to them alongside other strategies to address sex and gender factors that evidence suggests can assist women in achieving their treatment goals.

Many participants felt there was a need for clearer communication on the services the clinic offered. One method of improving communication that has implemented in our clinic since these focus groups has been placing a television in the waiting area that displays information about the clinic, including names and photos of the clinic staff, current services offered at the clinic, and how to access services and find out more information.

For several women, clinic location and hours of operation restricted level of participation in treatment. It is unknown whether women with barriers that prevented full participation experienced differential or poorer treatment outcomes or to what extent these factors may have prevented other women from initiating *any* treatment. Shifting hours of operation so that services are offered for fewer hours during the day but are available at least one weekday evening and/or for a half-day on the weekend may address this need. Offering individual counselling remotely may be the most practical way of overcoming this. Our clinic is currently developing a mobile application for smartphones to provide support for quitting smoking. This will help reduce barriers due to clinic hours and location for both current clients and also anyone who would like to quit smoking and has access to a smartphone.

Childcare was speculated to be important for other women, though no focus group participant stated that it would help them directly to have childcare available. An examination of demographic data from our clinic in the fiscal year prior to these focus groups does not suggest that fewer women were attending services compared to men in any age group except the 45–54 age range. While this does not rule out the fact that childcare responsibilities may hinder treatment seeking for women or men (including grandparents) with childcare responsibilities, it does highlight the need for further work to determine whether and to what extent availability of childcare would facilitate initial enrollment and subsequent level of participation in treatment.

Some of the themes that emerged here are consistent with findings in previous qualitative research. In one study, female veterans in the US also expressed a strong preference for both supportive services and having a choice of what services to use available when seeking smoking cessation treatment [25], confirming the importance of both of these features for women beyond the sample in the current study. Qualitative research with low-income women in Canada also identified several similar needs and preferences as reported here, including a “menu of support options” from which women could choose, child care onsite, free smoking cessation aids, and peer support opportunities (e.g., a “buddy”) [26]. Thus, the current study further validates the relevance of these recurring themes within women-specific program research and provides the groundwork for future research that can explore and rigorously test how adopting these program changes might translate into improved quit rates for women and whether they need to be further adapted to meet the needs of specific subpopulations of women who may experience unique challenges with quitting (e.g., psychiatric comorbidities, other addictions, etc.).

Some of these themes are also consistent with several principles of trauma-informed care that have been previously integrated into addiction services for women [27–29] based on evidence of the high rate of trauma history among this population [30]. For example, trauma-informed care aims to maximize a woman’s choice and control over treatment as well as work collaboratively with her to minimize any power imbalances so that treatment can be an empowering experience [27, 29]. Another principle of trauma-informed care is the creation of an environment for clients that is safe, respectful and accepting [29], with clear communication being one of several recommended strategies for enhancing safety. Thus, ours together with previous findings suggest that women regard the integration of these particular principles of trauma-informed care into treatment as beneficial and supportive. In fact, soliciting and incorporating feedback from clients in the design and evaluation of treatment services is another principle of trauma-informed care that helps to ensure the other principles are achieved [29].

## Limitations

This study has several limitations. The women that attended the focus groups may not be representative of all women who seek treatment in the clinic, or who seek smoking cessation services in general. The views expressed also cannot speak to the needs of women who wish to seek treatment but experience barriers to doing so. Though reflective of the demographics of the clients seen in the clinic, the majority of the sample was 50 years or older, thus the findings may not be applicable to younger women. Though both positive and negative aspects of the clinic were discussed, it is possible that some women may have not felt comfortable expressing their honest opinions, particularly if they conflicted with the views of other women in the group and given that the focus groups were conducted in the same building as the clinic. Another potential limitation is that the focus group questions were not derived on the basis of any theoretical framework. In addition, because there are no clear guidelines on how to determine the point at which saturation has occurred, it is possible that additional themes may have emerged with further sampling. Finally, another caveat to the current findings are that some of the same themes may emerge among men, and further research would be necessary to determine which of these needs and preferences are specific to women and which are applicable irrespective of gender.

## Conclusions

Evidence that men and women experience different rates of success with quitting [1, 2] and that sex (biology) and gender (social) both play important roles in smoking cessation [2] are important indicators that women need appropriately tailored programming. Results of the current evaluation help to shed light on what features of smoking cessation treatment meet the needs of female clients and may suggest an actionable list of adaptations that can be adopted by other clinics providing smoking cessation services to women, in addition to other evidence-based practices that address sex and gender differences undermining women’s success in treatment. An ideal program for women appears to be one that includes sufficient variety and choice to permit each woman to self-identify the particular combination of services that supports her own unique needs, flexible free pharmacotherapy, non-judgmental support, accessible services and clear communication of program options and changes.

## Additional file

Additional file 1: Focus Group Discussion Guide. (DOC 43 kb)
