# Supplementary material for: Woman focused smoking cessation programming: a qualitative study
Source: BMC Womens Health. 2016 Mar 12;16:17. doi: 10.1186/s12905-016-0298-2 (PMC4788944; doi:10.1186/s12905-016-0298-2)
Supplement: Additional file 1: — Focus Group Discussion Guide. (DOC 43 kb) [file 12905_2016_298_MOESM1_ESM.doc]

**Focus Group Discussion Guide**

Nicotine Dependence Clinic (NDC)

**INTRODUCTION**

- Introduce ourselves
- Why are we here today?
  - Differing points of view, no wrong answers, positive and negative comments welcome
  - Help inform future services
- Tape recorder and note taking
- First name basis today but no names will be in the report
  - Cannot guarantee confidentiality
  - Ask that no one repeat what was said here today or who attended
- Each of you will receive $25 for your time plus tokens if you came by TTC
  - Free to leave anytime
  - Washrooms
  - Evaluation form
- Consent form
- Questions

*Well, let's begin. Let's find out some more about each other by going around the table.*

***Icebreaker (5 minutes):***

1. **Tell us your name and what it means to you.**

***Transition Questions (20 minutes):***

1. **How did you hear about the NDC and why did you decide to come?**

***Key Questions (50 minutes):***

1. **If you were inviting a female friend to participate in the NDC, what would you say in the invitation? How about a male friend?**

- *Probe: What is good about the NDC for female clients?*
- *What are some of the barriers specific to female clients?*

1. **What has been most helpful to you about the services at the NDC?**

- *Write them on flipchart (assistant moderator) in point-form*
- *Keep topic on positive aspects before turning to negative aspects (if anyone brings a negative aspect up)*

1. **What changes would you like to see to the services offered at the NDC that would help you better overcome the challenges of quitting?**

- *Write them on flipchart (assistant moderator) in point-form*

Prompts/probes:

- Any topics for groups/workshops
- additional services (child care/ food/ etc)
- Online information or tools
- Take-home materials/readings
- Referrals to other services in the community

**a) Of all the suggestions we discussed, which are the top 3 you think would be most**

**important for you?**

- *group consensus – circle on flipchart (asst moderator)*

***Closing Question (15 minutes):***

1. **We asked you here today to help us know what difference the NDC is making for women trying to stop smoking and to help us know how to improve the services. Is there anything that we missed? Is there anything you wanted to say but didn’t get a chance to?**

***Reminder: Thanks for coming today. One last reminder to please keep this conversation confidential. We really appreciate your participation today.***
